# The roles of motion, gesture, and embodied action in the processing of mathematical concepts

**DOI:** 10.3389/fpsyg.2022.969341

**Published:** 2022-10-14

**Authors:** Omid Khatin-Zadeh, Danyal Farsani, Zahra Eskandari, Fernando Marmolejo-Ramos

**Affiliations:** ^1^School of Foreign Languages, University of Electronic Science and Technology of China, Chengdu, China; ^2^Department of Teacher Education, Norwegian University of Science and Technology, Trondheim, Norway; ^3^Facultad de Educación, Psicología y Familia, Universidad Finis Terrae, Santiago, Chile; ^4^Programa de Pós-Graduação em Educação Matemática, State University of São Paulo (UNESP), Rio Claro, Brasil; ^5^Department of English, Chabahar Maritime University, Chabahar, Sistan and Baluchestan, Iran; ^6^Center for Change and Complexity in Learning, The University of South Australia, Adelaide, SA, Australia

**Keywords:** motion, gesture, embodied action, perspective, frame of reference

## Abstract

This article discusses perspective and frame of reference in the metaphorical description of mathematical concepts in terms of motions, gestures, and embodied actions. When a mathematical concept is described metaphorically in terms of gestures, embodied actions, or fictive motions, the motor system comes into play to ground and understand that concept. Every motion, gesture, or embodied action involves a perspective and a frame of reference. The flexibility in taking perspective and frame of reference allows people to embody a mathematical concept or idea in various ways. Based on the findings of past studies, it is suggested that the graphical representation of a mathematical concept may activate those areas of the motor system that are involved in the production of that graphical representation. This is supported by studies showing that when observers look at a painting or handwritten letters, they simulate the painter’s or writer’s hand movements during painting or writing. Likewise, the motor system can contribute to the grounding of abstract mathematical concepts, such as functions, numbers, and arithmetic operations.

## Introduction

Embodiment theories and their implications have been the subject of an increasing trend of research in recent decades. A section of these studies focused on the role of gesture in teaching and learning (e.g., Goldin-Meadow and Wagner, 2005; Alibali and Nathan, 2007; Goldin-Meadow and Beilock, 2010; Atit et al., 2014; Nathan and Walkington, 2017; Alibali et al., 2019). According to McNeill (1992, 2008) typology, gestures are classified into four types: pointing gestures, iconic gestures, metaphoric gestures, and beat gestures. Pointing gestures are used to point to objects or locations. Iconic gestures depict the shapes of objects or their semantic content through the shape of hands or the trajectory of hand movements. Metaphoric gestures depict the meaning aspects of concepts indirectly through metaphors. In fact, metaphoric gestures depict the shape of the base domain of the metaphor (usually a concrete concept) to refer to the target domain of the metaphor (usually an abstract concept). For example, a pushing gesture can refer to the progress of a project (the metaphor *push the project forward*). Beat gestures do not depict semantic content of concepts but accompany speech as a rhythmic alignment. Iconic and metaphoric gestures are often put in one category and are called representational or depictive gestures (Alibali and Nathan, 2012). Representational gestures can be defined as gestures that literally or metaphorically depict the semantic content of concepts (Kita, 2000; Alibali et al., 2001). Throughout this paper, the word ‘gesture’ is used to mean representational gesture. Findings of many studies have demonstrated that gesture is an effective tool to enhance the processes of teaching and learning in various fields, including mathematics (Martinez-Lincoln et al., 2018; Walkington et al., 2019), physics (Johnson-Glenberg and Megowan-Romanowicz, 2017), astronomy (Lindgren et al., 2016), dynamic systems (Kang and Tversky, 2016), word learning (McGregor et al., 2009; Hupp and Gingras, 2016), math analogies (Richland and McDonough, 2010), story understanding (Beattie and Shovelton, 1999), and foreign language learning (Macedonia, 2014, 2019). Gesture can even enhance music processing in adults and infants (Phillips-Silver and Trainor, 2005, 2007). An important part of these research projects has been conducted within the framework of gesture elicitation studies (e.g., McAweeney et al., 2018; Smith and Gilbert, 2018; for a review, see Villarreal-Narvaez et al., 2020).

Motion is generally defined as the movement of an object against a background (Zlatev et al., 2012). Every motion is described relative to a frame of reference. There are three types of spatial frame of reference: viewpoint-centered (egocentric), geocentric, and object-centered (Zlatev, 2005, 2007; Zlatev et al., 2012). Within viewpoint-centered frame of reference, the object’s movement is described relative to the observer’s position (e.g., turn right). Geo-cardinal positions serve as the reference in a geocentric frame of reference (e.g., go to the south, raindrops are falling). In an object-centered frame of reference, the position of the moving object, or that of an external object, serves as the reference point (e.g., he entered the room). The differentiation between various types of frame of reference has some implications when concepts are described through the mediation of gestures. Gestures are defined as the spontaneously produced body movements accompanying our speech and thought (Goldin-Meadow, 2005; Chu and Kita, 2011; Nathan, 2014). DeSutter and Stieff (2017) define embodied actions as the purposeful and directed body states or body movements that an individual makes to learn something. These purposeful body states or body movements describe the spatial representations of concepts or relations between concepts. A very closely related concept is directed action. Thomas and Lleras (2009) define directed actions as body movements that students are instructed to engage in to learn a concept or to solve a problem. Directed actions can be seen as a subset of embodied actions. Throughout this article, we use ‘embodied action’ as a broad term that includes directed actions. The following two sections review some works on motion-based metaphors, gestures, and embodied actions to prepare the ground to show how these tools can be employed to enhance the process of mathematics teaching and learning.

## Spatial concepts, motion concepts, and motion-based metaphors

Many daily and scientific concepts are inherently motion events or are spatial in nature. For example, when we talk about a flying bird, we may show the trajectory of the movement by a hand gesture. When we talk about a circular object, we may use a hand gesture to show the shape of that object. Many concepts are metaphorically described in terms of motion events (e.g., Khatin-Zadeh et al., 2022a,b). The metaphor *we are approaching holidays* describes an abstract concept in terms of a motion event. The metaphorical phrase *grasp an idea* describes an abstract concept in terms of a body action. We may use gestures to describe these abstract concepts metaphorically. In mathematics, many concepts are metaphorically represented by spatial concepts or motion events (Lakoff and Núñez, 2000; Marghetis and Núñez, 2013). These metaphors are called mathematical metaphors. The role of mathematical metaphors in enhancing the process of mathematics learning has been supported by many works (e.g., Bazzani, 2001; Núñez, 2005, 2007; Edwards, 2009; Font et al., 2010).

Using a mathematical metaphor means mapping one representation of a mathematical concept (base representation) into another representation (target representation) and understanding the target representation in terms of the base representation. This is a metaphorical process as the former representation is structured and understood in terms of the latter representation (Lakoff and Núñez, 2000; Khatin-Zadeh, 2021). Mathematical metaphors and linguistic metaphors are inherently based on similar processes (Khatin-Zadeh, 2021). Both describe and represent a target concept in terms of a base concept. One justification for differentiation between mathematical metaphors and linguistic metaphors is the more substantial degree of the rigorousness of mathematical metaphors. The similarity or isomorphic relationship between the target and base of every mathematical metaphor is based on precise mathematical logic, making them more rigorous than linguistic metaphors. Lakoff and Núñez (2000) discuss the description of numbers in terms of points on a line, the description of functions in terms of curves in a Cartesian plane, and the description of functions in terms of fictive motions. A fictive motion metaphor describes a static concept in terms of a motion event (Talmy, 1996). That is, the feature of movement is fictively attributed to a static situation. The metaphors *f(x) never goes beyond 1,* and *f(x) oscillates between-1 and 1* are two examples in which a mathematical concept is metaphorically understood in terms of a fictive motion. Limit and continuity are two fundamental concepts in calculus that are conceptualized as dynamic concepts and fictive motions, although they have been formally defined in entirely static terms (Marghetis and Núñez, 2013). Crucially, these spatial concepts and fictive motions can be described by dynamic depictive gestures. Dynamic depictive gestures are gestures that show a motion-based representation of a concept through multiple body states (Nathan and Walkington, 2017; Pier et al., 2019). Describing abstract mathematical concepts in terms of concrete spatial concepts and fictive motions can help people ground these abstract concepts in easily perceivable concrete concepts (Khatin-Zadeh et al., 2021b). This is particularly the case when hand gestures are employed to show the shape of spatial concepts or the trajectory of the fictive motion.

The strong version of embodiment (Gallese and Lakoff, 2005) holds that understanding the target concept of a metaphor involves activating those neural networks that represent the base concept of that metaphor (for a review, see Khatin-Zadeh et al., 2021a). Therefore, the strong version of embodiment predicts that understanding fictive motion metaphors involves activating motor areas in the brain. This prediction is supported by the findings of several behavioral studies that suggest processing every fictive motion metaphor involves a mental simulation of the fictive motion (e.g., Boroditsky and Ramscar, 2002; Matlock, 2006; Núñez et al., 2006; Matlock et al., 2011). In one of these studies, Matlock (2004) conducted four experiments. In each experiment, participants read a story about travel (fast vs. slow, short vs. long distance, easy vs. difficult terrain). After reading the story, they decided on a fictive motion sentence. Overall, times of decision making were shorter after they had read about fast travel, short distances, and easy terrains. Such findings suggest that processing fictive motion sentences involves mental simulation of these motions. Matlock (2010) suggests that processing fictive motion metaphors involves experiencing a momentary sense of motion. The findings of a neuroimaging study suggest that a motor area in the brain that responds to motion perception (MT+) is activated during the processing of fictive motion metaphors (Saygin et al., 2010). According to the strong version of embodiment, the same cognitive resources that are employed while observing the oscillation of a moving object are also employed to process the mathematical metaphor *f(x) oscillates between-1 and 1*. This could happen not only in the process of fictive motion metaphors but also for visual processing of images that contain implied motion. For example, there is some evidence suggesting that visual processing of images that contain traces of brushstroke could activate sensorimotor cortical circuits (Sbriscia-Fioretti et al., 2013). Therefore, it can be hypothesized that the same thing can happen when someone processes the visual representation of a mathematical concept. For example, it may happen when someone processes the curve of a function (visual representation of a function in the Cartesian coordinate system). In Section 7, we discuss some evidence from past studies that support this hypothesis.

## Gesture and embodied action

Mathematics teachers and students regularly use gestures to present a visual description of the concepts and the relations between concepts (e.g., Flevares and Perry, 2001; Richland et al., 2007; Arzarello et al., 2008; Bieda and Nathan, 2009). Gestures facilitate speaking by helping speakers in the process of activating mental images (Wesp et al., 2001), by helping them to package information into easily expressible units (Kita, 2000; Kita and Davies, 2009), and by facilitating lexical access (Krauss et al., 2000; Ravizza, 2003). Gestures help listeners in the process of simulating actions that are expressed by speakers’ gestures (Alibali and Hostetter, 2010; see also Cevasco and Marmolejo-Ramos, 2013) and enhance memory by chunking information (Godøy et al., 2009).

DeSutter and Stieff (2017) make a distinction between embodiment and embodied action and say that embodied actions are purposeful body states or body movements that give a spatial representation of a concept. They add that while embodiment is a process in the brain, embodied actions are the physical antecedents of this brain-based process. Results of some studies have shown the effectiveness of embodied actions in enhancing learning. Goldin-Meadow and Beilock (2010) argue that embodied actions enhance thinking processes through foregrounding action in mental representation. This is particularly the case with spatial thinking. It has been suggested that imagery and mental processes that underlie spatial thinking (e.g., Kendon, 1994, 2004; Goldin-Meadow, 1999) can be enhanced through instructed embodied actions (DeSutter and Stieff, 2017). This proposal is confirmed by the findings of three studies that have examined spatial thinking of students in mathematics (Koschmann and LeBaron, 2002; Goldin-Meadow et al., 2009; Enyedy et al., 2015; Flood, 2018), engineering (Alibali et al., 2011), and chemistry (Stieff et al., 2016). In the study conducted by Goldin-Meadow et al. (2009), they manipulated gesturing while presenting a new mathematics lesson. They found that children who were required to produce correct gestures learned more than children required to produce partially correct gestures and children that were required to produce no gestures. This suggests that gestures could enhance not only the processing of old ideas but also the creation of new ones. Furthermore, this proposal is supported by the findings of a recent study on action memory (Kubik et al., 2020); results of this study indicated that successful memory retrieval of phrases containing a verb and ensuing enactment of that verb contribute to the future recall of those phrases.

## Gesture, embodied action, and embodied numerical cognition

Numerical processing is a special area of mathematical cognition that has been widely investigated by researchers. Campbell (2015) notes that when numbers are used as formal mathematical concepts and in a context-free manner, they are viewed as abstract. He adds that this is also the case with arithmetic operations because the results of operations are invariant regardless of numeral format or the referents of numbers. However, the findings of many studies suggest that numbers carry sensorimotor connotations (e.g., Lindemann et al., 2007; Loetscher et al., 2010; Fischer, 2018). The embodiment of numbers and arithmetic operations and the role of sensorimotor codes in number and arithmetic processing are important because our knowledge of quantities and magnitude depends on them (Lindemann and Fischer, 2015). That is why the embodiment of numbers and arithmetic operations has been the subject of a large body of research over the last two decades (e.g., Moeller et al., 2011; Ganor-Stern and Goldman, 2015; Patro et al., 2015; Rugani et al., 2015; Stapel et al., 2015; Wasner et al., 2015).

In one of the empirical studies conducted on the role of finger counting in numerical cognition, Sixtus et al. (2020) examined the numerical meaning of fingers that are based on the number of fingers raised or the ordinal position of fingers. In this study, participants received tactile stimulation on the fingertips of one hand. In the first experiment, participants had to name the number of fingers that had been stimulated. In the second experiment, they had to name the number of stimulations they had received on one fingertip. Results showed that participants were faster and more accurate in cases that the set of stimulated fingers corresponded to finger counting habits and also in cases that the number of stimulations matched the ordinal position of the stimulated finger. Another study conducted by Sixtus et al. (2017) examined the impact of finger posing on number comprehension. Participants of this study were exposed either to pictures of canonical finger postures (visual priming) or made the same finger postures (motor priming). After being exposed to one of these primes, they had to use foot responses to classify auditory numbers as smaller or larger than 5. Results of this study revealed that manually adopted postures had a significantly more priming effect on magnitude classifications. A priming study conducted by Di Luca and Pesenti (2008) investigated the impact of a masked priming on numerical comparative judgments. Results of this study revealed that numeral finger configurations used as unconsciously presented primes speeded up comparative numerical judgments. Participants responded faster and more accurately when primes were numerical than when primes were non-numerical. All these findings emphasize the critical role of the body, gesture, and embodied action in the processing of numbers. In Section 7, the potentially important role of the motor system in the processing of numbers, arithmetic operations, and functions is discussed. The following section discusses the factors that are involved in understanding mathematical concepts through motion-based metaphors, gestures, and embodied actions.

## Perspective, and frame of reference

When gestures or embodied actions are used to learn a concept or to acquire a better understanding of an idea, there are some factors that could affect the degree of effectiveness of gestures or embodied actions. The perspective (orientation) of the observer, the embodying agent who performs the gestures or embodied actions, and frame of reference are three of these factors. This is particularly the case in understanding mathematical concepts through motion-based metaphors. When a mathematical concept or idea is metaphorically described in terms of a motion event, depending on the observer’s perspective and frame of reference of the motion, the form of gestures or embodied actions varies. In other words, from a given perspective and within a given frame of reference, that concept or idea is described by a certain form of gesture or embodied action. An example could make the point clearer. The visual representation of a function may be shown as a curve in a Cartesian coordinate system. This curve can be understood as the trace of a fictive motion. This fictive motion has a starting point and an ending point. From one perspective, the observer can see the starting point and the ending point on her left and right, respectively. From an opposite perspective, the observer would see the starting point and the ending point on her right and left, respectively. However, from another perspective, the observer would see the starting point and the ending point along a hypothetical line that goes straightly into her eye. In fact, there is an infinite number of perspectives from which the fictive motion can be seen. Therefore, an algebraic representation of a function can be embodied in terms of a fictive motion in an infinite number of ways. The key point is that various perspectives could lead to a shared understanding of a concept. Understanding a concept from a certain perspective does not mean that the concept cannot be understood from other perspectives. In fact, an individual can understand a concept from a certain perspective and at the same time simulate that concept from other perspectives.

Beveridge and Pickering (2013) discuss perspective taking in spatial language and in action language. They note that in action language, the individual may adopt the perspective of the agent (performer of the action) or an observer. They add that perspective taking is flexible in spatial language, and this flexibility enables people to maximize the similarity between their mental models of a situation. Concerning frame of reference, Levinson (2003) distinguishes among three types of frame of reference: intrinsic, absolute, and relative frame of reference. In an intrinsic frame of reference, the position of every object is described relative to the position of a reference object (e.g., the intersection point is outside the circle). In an absolute frame of reference, the position of every object is described relative to arbitrary fixed environmental positions, which are independent of the viewer. For example, the village is to the south of the lake describes the location of the village independently of the position of the speaker or of any part of the lake. The relative frame of reference may be egocentric or allocentric. Egocentric perspective means seeing the objects from your perspective. Allocentric perspective means imagining seeing the objects from the perspective of someone else (for more details, see Levinson, 1996, 2003). Perspective and frame of reference affect the ways that concepts are embodied in the interactive context of a classroom. For example, a mathematics teacher may use the positions of his hands to show different types of angles (Alibali et al., 2014) or geometric shapes. These gestures and embodied actions can be used by students to acquire a better understanding of such concepts (Nathan et al., 2007; Yoon et al., 2014; Kelton and Ma, 2018). According to Kita et al. (2017), gestures and embodied actions may draw attention to a particular element of a concept or schematize certain features of a concept that is described in terms of a perceptual-motoric representation. This function of gestures and embodied actions is useful both for the producer and for the recipient of the gestures and embodied actions.

**Figure 1:**
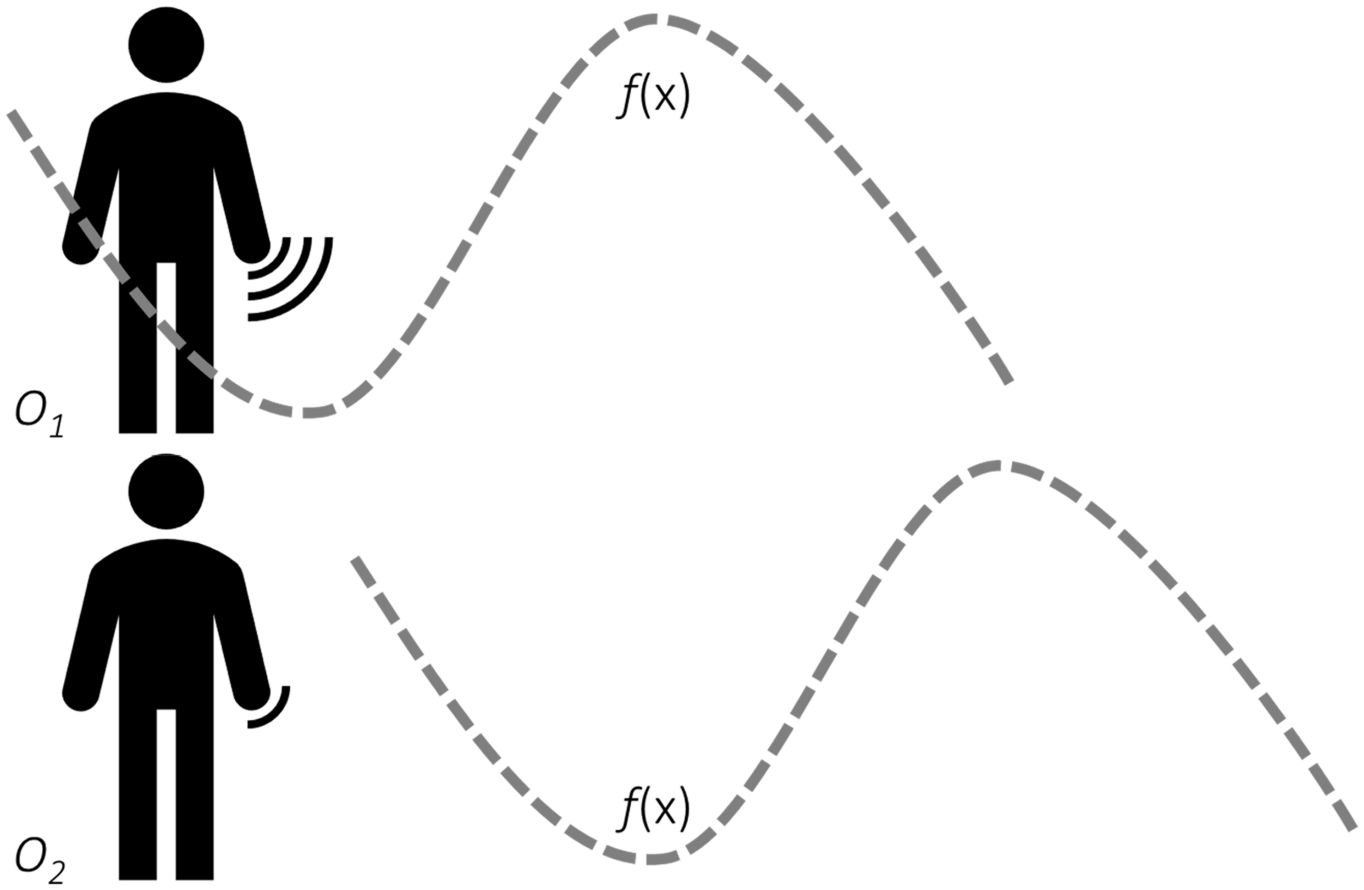
Illustration of the role of hand gestures for the understanding of mathematical concepts. *f*(x) stands for a mathematical function (e.g., *sin (x)*) that is amenable to visualization (in this case, a 2D Cartesian representation). *O_1_* represents an active observer (character’s viewpoint), and *O_2_* represents a passive observer (observer’s viewpoint). The interface icon by the hand of *O_1_* and *O_2_* illustrates the strength of ‘embodied resonance’ elicited in each scenario. *O_1_* has a stronger degree of engagement with the graph and imagination of the graph rather than seeing the graph (as it is the case with *O_2_)*.

When a teacher describes a concept through gesture, she creates the visual representation from her perspective within a certain frame of reference. The student sees the visual representation as an external observer. The formation of a mental representation of that concept takes place through a mapping process. In this process, the student maps the received representation into a new representation from her perspective within her frame of reference. In fact, this process is a mapping of perspective and a mapping of frame of reference. In an interesting study, Gerofsky (2010) examined the relationship between gesture’s perspective and students’ achievements in mathematics. She found an association between a character’s viewpoint of gesture and higher achievements in mathematics. Results of this study indicated that a character’s viewpoint of gesture was linked to a greater awareness of salient features of mathematical graphs, such as slope, roots, and extrema. That is, students who took a character’s viewpoint reported a more substantial degree of engagement and imagination of being the graph rather than seeing the graph (Figure 1). Based on these results, she suggested that such awareness led to a deeper understanding of the key features of polynomial functions.

In collaborative gestures and embodied actions, mapping of perspective and mapping of frame of reference becomes more complex, as two or several individuals with different perspectives and different frames of reference interactively create a model for understanding a single concept or idea. Although that concept or idea may be embodied differently from different perspectives and within different frames of reference, these different embodied representations are isomorphic with each other, as they represent a single concept or idea. Here, what is important is the embodied representation that is shared by all collaborators (with different perspectives and different frames of reference). As two or more people collaborate through gestures to create a visual representation of a concept or an idea, they have to create a shared frame of reference. Collaborators cannot share precisely the same perspective at the same time. However, this will not create any problem in acquiring a shared understanding of a concept or an idea. The following section discusses perspective and frame of reference during the activation of affordances of objects.

## Perspective and frame of reference in affordances activation

The concept of affordance was initially introduced by Gibson (1979). He defines affordances as action possibilities that are provided by the environment. When we look at objects in our surrounding environment, we see that every object gives us the opportunity to perform some kind of action on it. For example, a glass is a graspable object; thus, it gives us the opportunity to grasp. Likewise, we can walk on a road; thus, it gives us the opportunity to walk on. According to Borghi (2018), affordances are the opportunity of actions that are offered by the environment to an agent who has the potential to perform those actions. Reed (1996) views affordances as properties of an object or the environment relative to an agent. Chemero (2003, 2009) holds that affordances are, in fact, relations between certain aspects of the agent and certain aspects of the environment. Some views in cognitive science hold that affordances are the result of long-term visuomotor associations and are activated automatically (Borghi, 2018). For example, observing a graspable object leads to the activation of those neural substrates involved in the actual grasping of that object (Rizzolatti and Craighero, 2004; Gallese and Lakoff, 2005; Rizzolatti and Fabbri-Destro, 2008; Hess and Fischer, 2013). Several empirical studies have provided evidence that suggests affordances may automatically be activated (e.g., Ellis and Tucker, 2000; Tucker and Ellis, 2001; Phillips and Ward, 2002; Grèzes et al., 2003; Vingerhoets, 2008, 2014; Bloesch et al., 2012; Kourtis et al., 2018). However, the automatic activation of affordances has been challenged by the findings of studies that have found evidence suggesting that activation of affordances is task-dependent (e.g., Bub et al., 2018; Xiong et al., 2019; Chong and Proctor, 2020). Therefore, when we look at objects that afford motion or have a kind of relationship with body movements, the motor system could be activated, although this activation may depend on the task that we are going to perform. This could happen not only when we look at those objects but also when we think about them.

If looking at or thinking about an object (or its metaphorical representation) activates the motor system, it can be said that a motion event is simulated in the mind of the comprehender. This motion simulation can be done from various perspectives and within a variety of frames of reference. When an observer looks at an image showing a running man, the action of running itself or the process of observing a real running man can be simulated in the mind of the observer. In other words, the observer of the image can imagine himself as the runner or as someone who looks at a running man. This simulation can be done from a variety of perspectives. Even observing the image of a road may activate the motor system, as a road affords movement. People can walk, run, and perform many body movements on a road. In the same way that looking at a glass or the image of a glass may activate those neural substrates that are involved in the action of grasping, observing a road or the image of a road may activate those neural substrates that are involved in walking or running on a road. In more complex cases, thinking about highly abstract mathematical concepts that are metaphorically described in terms of graphical representations can activate the motor system. In such cases, it may be said that those hand movements that have produced the graphical representation of that abstract concept are simulated. The following section discusses the findings of several empirical studies that support this proposal.

## Simulating hand movements that produce a graphical representation

As proposed, when we describe a mathematical concept in terms of a graphical representation and think about that mathematical concept in terms of its graphical representation, those hand movements that have produced the graphical representation are simulated. For example, when the function *f(x)* is described in terms of a graphical representation and described metaphorically as *f(x) oscillates between-1 and 1*, the oscillatory movement could be simulated in the mind of the comprehender. From the perspective of the strong version of embodiment (Gallese and Lakoff, 2005), the same areas in the brain involved in the processing of a real oscillatory movement are also involved in the processing of this function. This proposal is supported by studies showing that looking at a painting may lead to simulating those hand movements that are involved in the production of that painting. In one of these studies (Umilta' et al., 2012), neural activities were examined during observing Lucio Fontana’s paintings. The findings of this study suggested that during observing abstract works of art that include some kind of implied motion, the cortical motor system was activated. Another EEG study (Sbriscia-Fioretti et al., 2013) examined the involvement of sensorimotor cortical circuits when an observer looked at images of abstract works of art with marked traces of brushstrokes. Results of this study indicated that premotor and motor cortical areas were activated when the observer looked at these works. Gallese and Sinigaglia (2011) propose that observing a cut in a canvas could activate the cortical motor system. A related group of studies has specifically focused on the visual perception of letters. It has been found that looking at static letters can activate cortical motor areas (Longcamp et al., 2003; James and Gauthier, 2006). Longcamp et al. (2011) compared the neural correlates of perceiving handwritten letters vs. printed letters. They found that visual perception of handwritten letters involved a stronger activation in the left primary motor cortex and the supplementary motor area. The strong activation of motor areas during perceiving handwritten letters could be the result of simulating those hand actions that produce the handwritten letters. If looking at abstract works of art or static letters can lead to the simulation of those hand movements that have produced them, it could also be the case with looking at a graphical representation of mathematical concepts and thinking about the graphical representation of mathematical concepts (Khatin-Zadeh et al., 2022c). Therefore, the motor system can contribute to the grounding of even highly abstract mathematical concepts. The motor system can also be employed in the processing of numbers and arithmetic operations. Since numbers can be represented on an axis, arithmetic operations can be understood as movements on the axis. For example, the equation (−2) + (−5) = (−7) can be understood as a movement from −2 to −7. In fact, a combination of finger counting and hand movements can be used to process numbers and arithmetic operations. In this way, the simulation of movements and the motor system could play a role in the processing of numbers and arithmetic operations.

Here, an important point must be noted. Since graphical representation of a concept can logically be the product of an infinite set of hand movements (as it is a running simulation), the comprehender may divide the graphical representation into a set of small parts and simulate each part in different stages. The division could be based on where the key features of graphical representation are located. For example, the local maximum point of the graphical representation of a function can be put in a small separate section. The simulation of those hand movements that produce this small part can help the comprehender acquire a better understanding of the local maximum point. Therefore, if a key feature of a concept is placed in one separate part of its graphical representation, the simulation of those hand movements that produce that part could help the comprehender acquire a deeper understanding of that specific feature. Manipulating some points of a graphical representation by gesture is a technique that can be used to foster the process of learning mathematical concepts. Moving a parabola along the X or Y axis in the Cartesian coordinate system, changing the parameters of the equation, and choosing another origin are some exercises that can be supported by gestures to help students acquire a deeper understanding of the equation of a parabola and its graphical representation in the Cartesian coordinate system. Châtelet (2000) argues that the gestural and the graphical representations of mathematical concepts are pivotal sources of mathematical meaning. He adds that it is impossible to separate immovable mathematics (static representations of mathematical concepts) from movable matter (dynamic representations of mathematical concepts such as gestures). Similarly, de Freitas and Sinclair (2011, 2014) argue that any attempt to separate mathematics from the material world (embodied or material representations of mathematical concepts) is just an idealistic view.

## Conclusion

The aim of this article was to show how motions, gestures, and embodied actions contribute to the grounding of abstract mathematical concepts and enhance the process of mathematics teaching and learning. When a mathematical concept or idea is metaphorically described in terms of gestures, embodied actions, or a fictive motion, the motor system comes into play to ground and understand that concept or idea. This mapping process, in which an abstract concept or idea is metaphorically represented in terms of body actions and movements, can facilitate the process of understanding. It was suggested that the graphical representation of a mathematical concept may activate those areas of the motor system that are involved in producing the graphical representation. This is supported by the findings of studies that have revealed that looking at a painted image or handwritten letters can be the cause of simulating those hand movements that are involved in painting that image or writing the letters. This sort of findings emphasize the important role that is played by motions, gestures, embodied actions, and the motor system in the processing of key mathematical concepts such as functions, numbers, and arithmetic operations. Although numerous studies have been conducted in this area of research, it seems that we have a long way ahead to acquire a deeper understanding of various aspects of embodied mathematical cognition. Specifically, direct neuroscientific evidence in support of our theoretical claim is in order.

## Data availability statement

The original contributions presented in the study are included in the article/supplementary material, further inquiries can be directed to the corresponding author.

## Author contributions

OK-Z wrote the first draft of this manuscript. DF, ZE, and FM-R: commented on it and revised it. All authors contributed to the article and approved the submitted version.

## Conflict of interest

The authors declare that the research was conducted in the absence of any commercial or financial relationships that could be construed as a potential conflict of interest.

## Publisher’s note

All claims expressed in this article are solely those of the authors and do not necessarily represent those of their affiliated organizations, or those of the publisher, the editors and the reviewers. Any product that may be evaluated in this article, or claim that may be made by its manufacturer, is not guaranteed or endorsed by the publisher.
